# Optical control of single-atom dynamics in plasmonic nanogaps

**DOI:** 10.1126/sciadv.adx3216

**Published:** 2025-07-18

**Authors:** Paul Kerner, Rakesh Arul, Damien Thompson, Jeremy J. Baumberg, Bart de Nijs

**Affiliations:** ^1^ Nanophotonics Centre, Department of Physics, Cavendish Laboratory, University of Cambridge, Cambridge CB3 0US, England, UK.; ^2^ Department of Physics, Bernal Institute, University of Limerick, Limerick V94 T9PX, Ireland.; ^3^ Physics for Sustainable Chemistry, Department of Physics, University of Cambridge, Cambridge CB3 0US, England, UK.

## Abstract

Observing and controlling dynamics of single atoms in ambient conditions is challenging when using conventional atomic-scale techniques due to their invasive character. Here, such control is achieved optically, by confining pulses of visible light within extreme plasmonic nanogaps, where they rapidly create (“write”) an adatom on one facet surface. Such adatoms are shown to be storable in ambient conditions for at least a week in the dark and are observed (“read”) using low-intensity surface-enhanced Raman spectroscopy (SERS). Writing at higher optical intensities stabilizes the atomic protrusion through light-induced local restructuring, which imposes a higher energy barrier for its return into the metal surface. Fluctuations in these “picocavity” SERS spectra show that while adatom movement is significantly slower under low light intensities, ambient thermal energy still enables them to explore the surrounding energetic landscape. Optical control over single metal atom dynamics opens promising avenues for next-generation microelectronics, atomic-scale imaging, and catalysis.

## Teaser

Visible light is used to quickly “write” an atom on a gold surface that can be stored and “read” for over a week.

## INTRODUCTION

Observing and controlling the dynamics of a single atom on a metal surface is not only of fundamental interest but also highly sought after within micro- and nano-electronics (*1*–*3*) due to the ever-stronger push for miniaturization. Devices involving single-atom electronical components (*4*) and memory (*5*, *6*) are of particular interest. Understanding the behavior of metal surfaces and their interaction with molecules in operando conditions is also key for catalysis (*7*–*9*), where single metal atoms are found to play increasingly important roles (*10*–*14*).

Traditional tools visualizing at the atomic scale are electron (*15*, *16*), scanning tunneling (*17*), and atomic force (*18*) microscopies, which are unfortunately often highly disruptive to delicate single-atom dynamics. An alternative solution is offered by tip-enhanced (*19*–*21*) or surface-enhanced (*22*–*25*) Raman spectroscopies (TERS or SERS) to probe systems down to single atoms. These use low-energy visible light, work in ambient conditions, and can access nanoscale dynamics (*26*). Single-atom resolution can now be achieved using extreme plasmonic light confinement on subnanometer scales around individual metal adatoms (termed picocavities), which interact with nearby single molecules, as recently modeled in detail (*8*, *27*–*31*). Picocavities have been successfully used for single-molecule sensing and spectroscopy (*32*–*35*) as well as understanding catalysis (*8*, *12*, *36*). Picocavity SERS can be easily accessed through straightforward and reproducible nanofabrication using self-assembled architectures such as the nanoparticle-on-mirror (NPoM), which enables diverse measurement strategies (*37*–*42*).

Picocavities have also been recently used to capture atomic-scale dynamics (*24*, *25*, *28*, *43*–*45*), revealing the complex energy landscape that a molecule-coordinated adatom inhabits on a metal surface. Light appears to play a critical role in enabling the adatom to explore this landscape by lowering the energy barriers for formation, diffusion, and decay (*24*, *28*). These results indicate the potential for deterministic control over the motion of single atoms (*8*), for instance, by tuning incident laser intensity, color, and irradiation time.

Here, we expand the understanding of adatom dynamics by demonstrating control over the formation, evolution, and decay of adatom systems through varying the illumination intensity in real time, working across the extreme ranges of μW to mW μm^−2^ illumination intensities compared to previous work (*22*–*25*, *28*). Furthermore, our data show that picocavities are robust and stable phenomena in the absence of illumination.

## RESULTS

### Plasmonic nanogap design

**Fig. 1.**
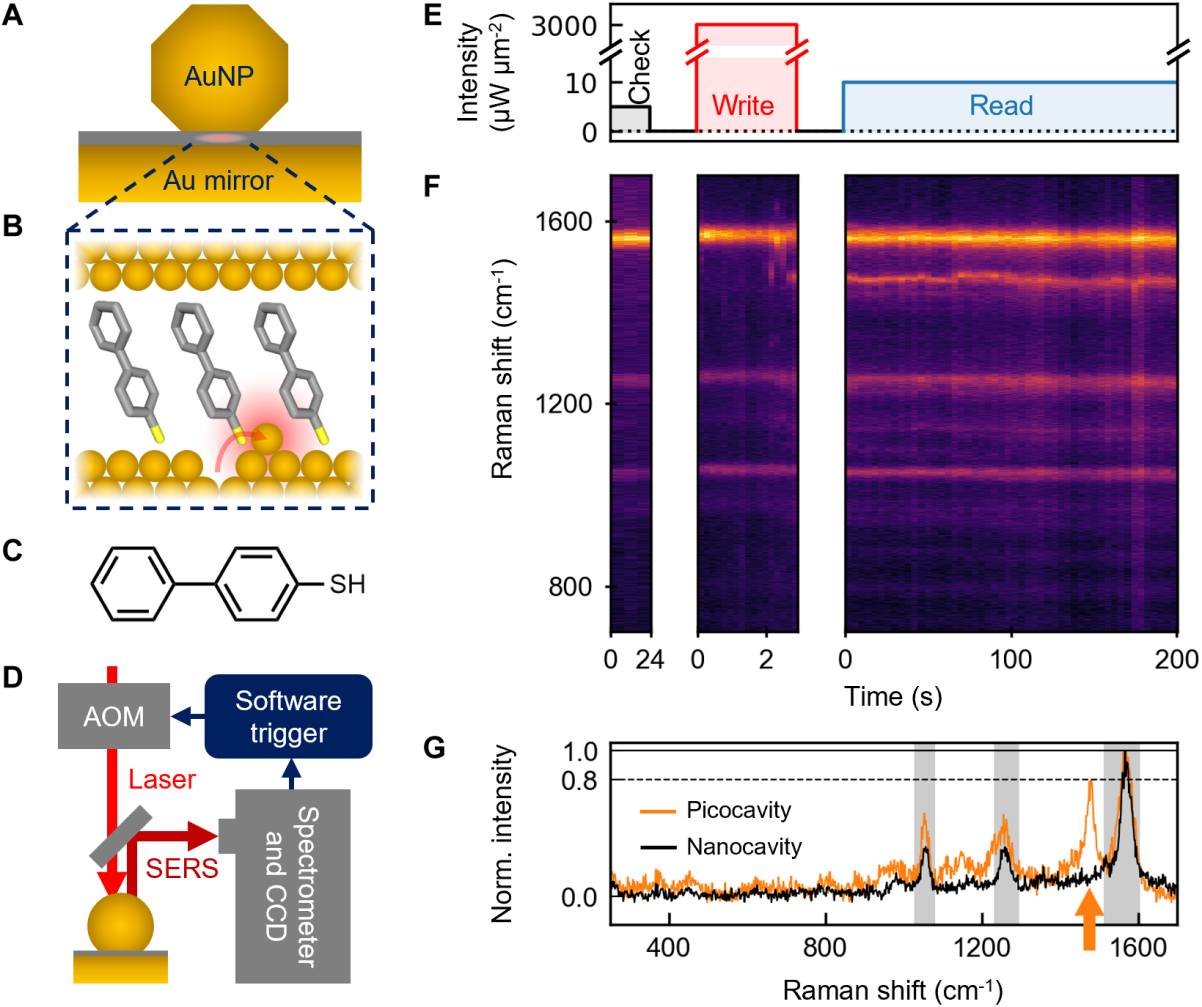
Writing and reading an adatom with light. (**A**) Nanoparticle-on-mirror (NPoM) structure, forming a nanoscale plasmonic hotspot (red) within a biphenyl-4-thiol (BPT) self-assembled monolayer (SAM) (gray). (**B**) Schematic of NPoM nanogap, inside which a gold adatom creates a picocavity that perturbs one BPT molecule (within its <1 nm^3^ optical mode volume, shaded). (**C**) Structure of BPT molecule. (**D**) Schematic setup used to write picocavities. Collected surface-enhanced Raman scattering (SERS) spectra are scanned every 170 ms for picocavities using charge-coupled device (CCD) camera. Upon picocavity detection, an acousto-optic modulator (AOM) lowers the irradiation intensity to near zero. (**E**) The 633-nm laser irradiation intensity over the course of a typical write-read experiment (note y scale). (**F**) Corresponding SERS time scans for (E). Initial check scan collected at 5 μW μm^−2^ with 8-s integration time per spectrum, write at 3 mW μm^−2^ for 170-ms irradiation time, and read at 10 μW μm^−2^ every 4 s (further scans in fig. S1). (**G**) Example of background-subtracted picocavity and nanocavity spectra normalized to the most intense nanocavity peak, used by the picocavity detection algorithm (triggers as “written” when any peak outside those shaded exceeds 80% of the normalized intensity, dashed line).

To access subnanometer light confinement, we create plasmonic nanogaps within NPoM constructs (Fig. 1A). These are constructed from a smooth gold mirror substrate coated with a self-assembled monolayer (SAM; Fig. 1B) of biphenyl-4-thiol (BPT; Fig. 1C). BPT is used for its ability to form high-quality SAMs due to π−π stacking (*46*, *47*), give strong Raman signals, and support reliable picocavity formation (*22*, *23*, *28*). Immersing the SAM-coated mirror in colloidal 80-nm gold nanoparticle (AuNP) suspension deposits millions of NPoM structures, each with several hundred BPT molecules sandwiched in the strong optical “hotspot” within each nanogap under the AuNP facet (*37*, *48*). Each NPoM construct acts as a nanoantenna, coupling light in and out of its nanogap. The small gap size of 1.3 nm (determined by ellipsometry) allows charges in the AuNP to couple to their images in the mirror and laterally confines light to ~5 nm, resulting in an optical nanocavity with intensity enhancements of >10^5^ (*38*) and SERS enhancement factors (EFs) of >10^8^ (*49*) in the nanogap.

### Picocavity SERS

Measuring SERS of NPoMs over time (Fig. 1F) results in stable peaks originating from the ~200 BPT molecules in the strong nanocavity field region of the nanogap (black spectrum in Fig. 1G) (*37*). This is termed the nanocavity SERS spectrum and does not change with time. Under illumination above a critical intensity of $I_{c}$~100 μW μm^−2^ for BPT (*28*), intense transient spectral features are typically observed (Fig. 1F). In this work, we concentrate on fleeting narrow-band features termed “picocavities” (red, Fig. 1G), which have been attributed to single Au^(0)^ adatoms lifting out of the gold surface within the nanogap (*22*, *23*, *27*). The adatom provides an additional picocavity field confinement with measured mode volumes <1 nm^3^ resulting in an additional Raman enhancement of ~100, thus allowing SERS from single molecules to become visible in the vicinity of the atomic-scale protrusion (*27*, *30*). Density functional theory (DFT) simulations show that the undercoordinated adatom interacts with the affected molecule (*23*, *46*, *50*, *51*), shifting its vibrational levels (*24*) and activating normally Raman-inactive modes due to the high-field gradient (*22*, *52*). Picocavities also induce vibrational pumping effects within the molecule, resulting in abnormally high anti-Stokes emission that scales quadratically with illumination power (*22*, *23*), which allows direct measurement of the optical volume ( < 1 nm^3^). Transient elevated anti-Stokes (equivalent to T≤ 2000 K) is not seen in the nanocavity spectrum, which remains unchanged, suggesting that chemical changes in the gap are not involved. Since typically only one perturbed vibrational spectrum is observed, from a single molecule coupling to the Au protrusion, a single Au adatom is strongly implied. Photochemical changes of the ~200 gap molecules instead manifest as a gradual onset of broad spectral features (*53*). In all cases, no picocavity SERS is seen initially, implying that the Au facets have no adatoms at the start. Simulations also show that the pit from which each adatom originates has little light confinement (*54*).

### Adatom dynamics

Picocavity formation and decay rates have been shown to greatly accelerate in the presence of strong illumination (*28*). As a result, picocavities preferentially form near the facet center where the optical fields are strongest (*25*). This has been explained by the local picocavity field further polarizing the nearest atom of the molecule, inducing dipole-dipole forces, and thus lowering the activation barrier to pull an adatom out (*28*), or back in. Adatoms form by overcoming this barrier at a stochastic rate from ambient thermal energy. The rate scales with the polarizability or electron-donating character of the nearest molecule moiety (*55*). Using this activation barrier lowering model, the picocavity formation rate $\Gamma_{W}$ is related to the incident light intensity *I*_W_ via

$$\begin{array}{c} \Gamma_{W}\left(I_{W}\right)=\Gamma_{0W}\text{exp}\left\{-\frac{U_{f}^{0}}{\left(1+I_{W}/I_{t}\right)k_{B}T}\right\} \end{array}$$ (1)

where $\Gamma_{0W}$ is a constant, $U_{f}^{0}$ is the formation barrier in the dark, $I_{t}$ is a threshold intensity, T is the temperature, and $k_{B}$ is the Boltzmann’s constant. The critical formation intensity $I_{c}∼I_{t}U_{f}^{0}/k_{B}T$ is defined as the light intensity where $\Gamma_{W}\left(I_{c}\right)=\Gamma_{0W}e^{-1}$ . Time variations in the adatom-molecule configuration manifest as intensity and frequency fluctuations of the picocavity lines, not observed for SERS lines from the unperturbed nanocavity (Fig. 1F) (*24*, *35*, *50*, *51*).

### Writing and reading picocavities

Because of this optical modulation of the activation barrier, picocavities once formed are found to be more stable at lower illumination intensities (*22*) while forming and decaying quickly at higher intensities (*28*). This opens up avenues for more deterministic control over these atomic features by controlling the irradiation power density. To this end, a series of automated SERS experiments are performed on each of hundreds of NPoMs, where the 633-nm laser power is varied to modulate picocavity formation and decay. Three stages of measurements are performed on each NPoM: an initial “check,” a “write” stage where a picocavity is formed, and a “read” stage that monitors the longevity of the formed atomic protrusion (Fig. 1, E and F, and fig. S1). The check stage consists of one to three spectra at <10 μW μm^−2^ to confirm a typical stable nanocavity, with the laser power density too low to create picocavities. During the write stage, an acousto-optic modulator (AOM) is used to deliver brief bursts (0.2 s) of more intense radiation (1 to 5 mW μm^−2^). Between each irradiation, SERS spectra are inspected for intense non-nanocavity features. Upon detecting that a previously absent feature exceeds 80% of the intensity of the highest nanocavity peak (Fig. 1G), a software trigger immediately ends the write stage. Finally, the read stage consists of a lower intensity (5 to 80 μW μm^−2^) time scan over 200 s to observe the stability of the written picocavity over time. Variations of this write-read procedure are performed on over 1000 NPoMs at different incident intensities to determine their effect on write speed and read survivability (see notes S2 to S4).

### Writing statistics

**Fig. 2.**
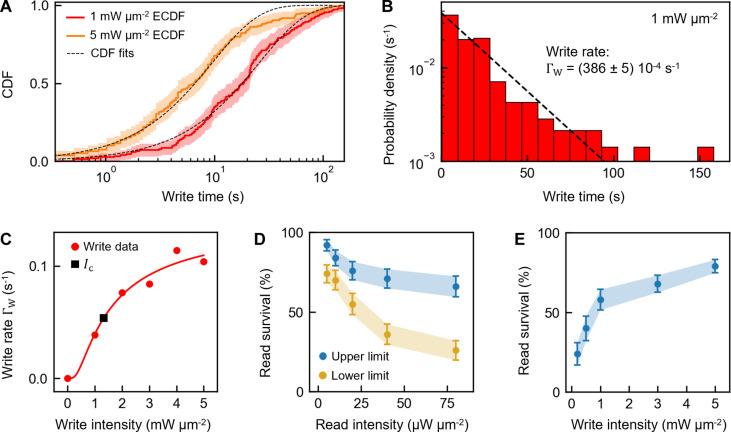
Picocavity writing and reading dynamics versus laser power density. (**A**) Empirical cumulative density distribution functions (ECDFs) of picocavity write time distributions along with exponential CDF fits ( $F_{W}=1-e^{-\Gamma_{W}t}$ ) for 1 mW μm^−2^ and 5 mW μm^−2^ incident laser power densities (dashed). Shaded regions denote 95% confidence intervals. (**B**) Histogram of picocavity write times at 1 mW μm^−2^ write intensity, with exponential probability distribution function (dashed line, $f_{W}=\Gamma_{W}e^{-\Gamma_{W}t}$ ) derived from the ECDF in (A). (**C**) Picocavity write rate $\Gamma_{W}$ versus laser intensity with fit to Eq. 1 model assuming 1-eV activation barrier (*28*); ◼ marks critical intensity $I_{c}$ . Error bars derived from CDF least squares fits are too small to be seen. (**D**) Percentage of picocavities that survive for 200 s versus read illumination intensity (at write intensity 3 mW μm^−2^). Upper and lower limit survival percentages extracted from criteria detailed in Materials and Methods and note S1. (**E**) Picocavity % survival during read versus laser intensity in the preceding write (read intensity 40 μW μm^−2^). Error bars in [(D) and (E)] are derived from binomial distribution variance.

Picocavity formation characteristics are determined using write-read experiments at varying write intensities, evaluating 150 to 200 NPoMs per intensity. Picocavity write times are directly extracted from the number of 170-ms write bursts before the trigger. Their distribution appears exponential, in agreement with previous results (*28*) (Fig. 2B). Fitting the exponential cumulative distribution function ( $F_{W}=1-e^{-\Gamma_{W}t}$ ) to the empirical cumulative density distribution function (ECDF; Fig. 2A and fig. S4) yields write rates $\Gamma_{W}$ . These scale with illumination intensity, continuing the trend in (*28*) to higher intensities (Fig. 2C). ECDFs show that ~50% of all picocavities are written within 5 to 20 s depending on intensity, demonstrating on-demand picocavity creation with 0.17-s time resolution. We find that $I_{W}$  ~ 3 mW μm^−2^ is optimal for best writing speed and mitigating irradiation damage to the system. We use Eq. 1 to fit the write rate results (Fig. 2C). Assuming that $U_{f}^{0}\;∼$ 1 eV (as discussed below) results in a critical intensity $I_{c}=$ 1.3 mW μm^−2^, an order of magnitude above the previously reported value for BPT (*28*). This difference is not unexpected since our protocol selects only the most intense picocavities (with >80% of the nanocavity intensity), thus increasing the optical intensity required to write one in each time interval.

### Reading picocavities

An optimal write power density (3 mW μm^−2^) is used to perform a set of write-read experiments to determine the stability of written picocavities against prolonged read irradiation, measuring 60 to 80 NPoMs for each read intensity. Read scans lasting 200 s are evaluated for whether the written picocavity survives until their end, yielding lower and upper limit survival rates at each read intensity (see Materials and Methods and note S1). Lowest read intensities (5 to 10 μW μm^−2^) yield the highest survival rates (75 to 90%), making them optimal for noninvasive reading (Fig. 2D and table S1). Increasing read intensity gradually decreases the survival rates, but at least 20% still survive for >200 s. This contrasts with previous results using constant light intensities for both formation and decay, where almost all picocavities decayed within 100 s (*28*).

Repeating these read-survival experiments for different write intensities (0.2 to 5 mW μm^−2^), constant read intensity of 40 μW μm^−2^ surprisingly shows that high write intensities result in more stable picocavities (Fig. 2E and table S2). There is a notable reduction in read survival rate below 1 mW μm^−2^ write intensities. This suggests that higher write intensities influence the eventual stability of picocavities formed. We suggest that this is the result of more extensive photo-induced surface restructuring resulting in the adatom-molecule finding a more stable state (see simulations below). This would also explain the anomalously high survival rates observed during read scans after writing at 3 mW μm^−2^ (Fig. 2D). Such surface restructuring could be induced by optical forces or light-induced thermal annealing, with previous work using different molecules in the gap implying that thermal effects are not dominant (*28*).

### Long-term dark storage

**Fig. 3.**
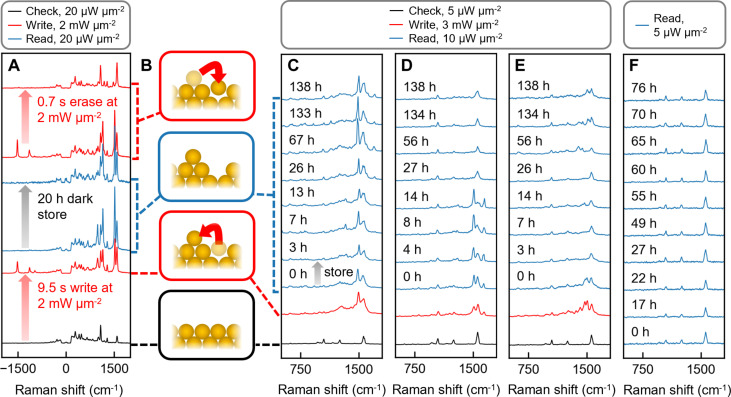
Picocavity long-term storage in darkness. (**A**) The 785-nm excitation SERS spectra resulting from a write-read-erase experiment with a 20-hour storage period at the read stage. Only the final write spectrum is shown. All spectra are normalized to the 290 cm^−1^ nanocavity peak and smoothed with a third-order 5-point Savitzky-Golay filter. (**B**) Schematic representations of adatom surface configurations corresponding to the spectra in (A) and [(C) to (E)]. (**C** to **E**) The 633-nm excitation SERS spectra resulting from write-read experiments on three example picocavities over the course of a week, with increasing periods of dark storage between read spectra. Only the final write spectra (red) are shown. (**F**) The 633-nm excitation SERS spectra resulting from read experiments on a control NPoM with no prior write stage, over the course of 76 hours. All spectra in [(C) to (F)] are normalized to the 1050 cm^−1^ nanocavity peak and smoothed with a third-order 11-point Savitzky-Golay filter. h, hours.

The read dynamics (Fig. 2D) show that below 20 μW μm^−2^ the chance of destroying a picocavity is low enough to allow repeated reading of picocavity states without substantial risk of decay, especially when reading for shorter times. In the absence of light, it should thus be possible to “store” these atomic-scale complexes for extended periods of time, even at ambient conditions. To test this, a proof-of-principle write-read experiment is performed on one picocavity at 785-nm excitation. The latter is used to bring anti-Stokes emission into resonance (*41*); this can confirm the presence of a picocavity from its distinctive vibrational pumping (*22*, *23*), giving short-wavelength Raman peaks for mW μm^−2^ intensities (Fig. 3A). The picocavity written at 2 mW μm^−2^ is followed by an initial 8-s reading at 20 μW μm^−2^. After writing the picocavity, the illumination is fully switched off, and only after 20 hours in ambient conditions is the picocavity state read out again, revealing a nearly identical picocavity spectrum (main picocavity line shifts <10 cm^−1^). This confirms that picocavities can be successfully “stored” in ambient but dark conditions for extended periods of time. Subsequently, the picocavity is again illuminated at 2 mW μm^−2^ and vibrational pumping is again clearly observed in the anti-Stokes spectrum (Fig. 3A), thus ruling out irreversible photochemical changes as the origin of the additional stable-in-darkness peaks. After < 1 s of 2 mW μm^−2^ illumination, the picocavity is “erased,” as evidenced by the disappearance of vibrational pumping in the anti-Stokes spectrum. We note that since the vibrational pumping has a quadratic power density dependence, it cannot be observed during lower intensity reads (*22*).

For analyzing storage lifetimes and behavior, a further 32 picocavities in separate NPoMs are written at 3 mW μm^−2^ with 633-nm excitation, and read in 4-s-long 10 μW μm^−2^ scans over the course of a week (138 hours), with increasing periods without illumination between spectra. Overall, ~10% of the picocavities survived for the full 138 hours while exhibiting peak shifts of < 20 cm^−1^ (Fig. 3C and fig. S5, A to C). Another 75% also survive throughout the week, but exhibit stronger spectral evolution with peak shifts of >20 cm^−1^ and peak (dis)appearance (Fig. 3E and fig. S5, D to H). The remaining picocavities decayed before the end of the experiment (Fig. 3D and fig. S5, I to M). Both evolution and decay can be the result of the sporadic read illumination, as these have been shown to occasionally happen even at such low irradiation intensities (Fig. 2D) (*28*). A control experiment performed on an unperturbed NPoM (omitting the write stage) is probed by 8-s-long read scans (5 μW μm^−2^) every few hours. This shows no picocavities forming over the course of 76 hours (Fig. 3F), implying a negligible write rate at ultralow illumination and that the picocavities observed above during reads are the originally written picocavity. These results show that picocavities can clearly be stored in dark conditions for at least 1 week. This length of time approaches the overall NPoM construct stability in air (*56*), signaling that picocavities are surprisingly robust entities.

### Spectral fluctuations

Picocavity spectral wandering is linked to changes in adatom-molecule configuration. SERS time scans of picocavities often show metastable states, where the picocavity switches between a set of similar spectral profiles over time (*24*, *35*, *50*, *51*). This implies that the adatom switches between local energy minima on the complex surface-molecule energy landscape. Gradual changes likely consist of many fast state switches that result in an average movement.

**Fig. 4.**
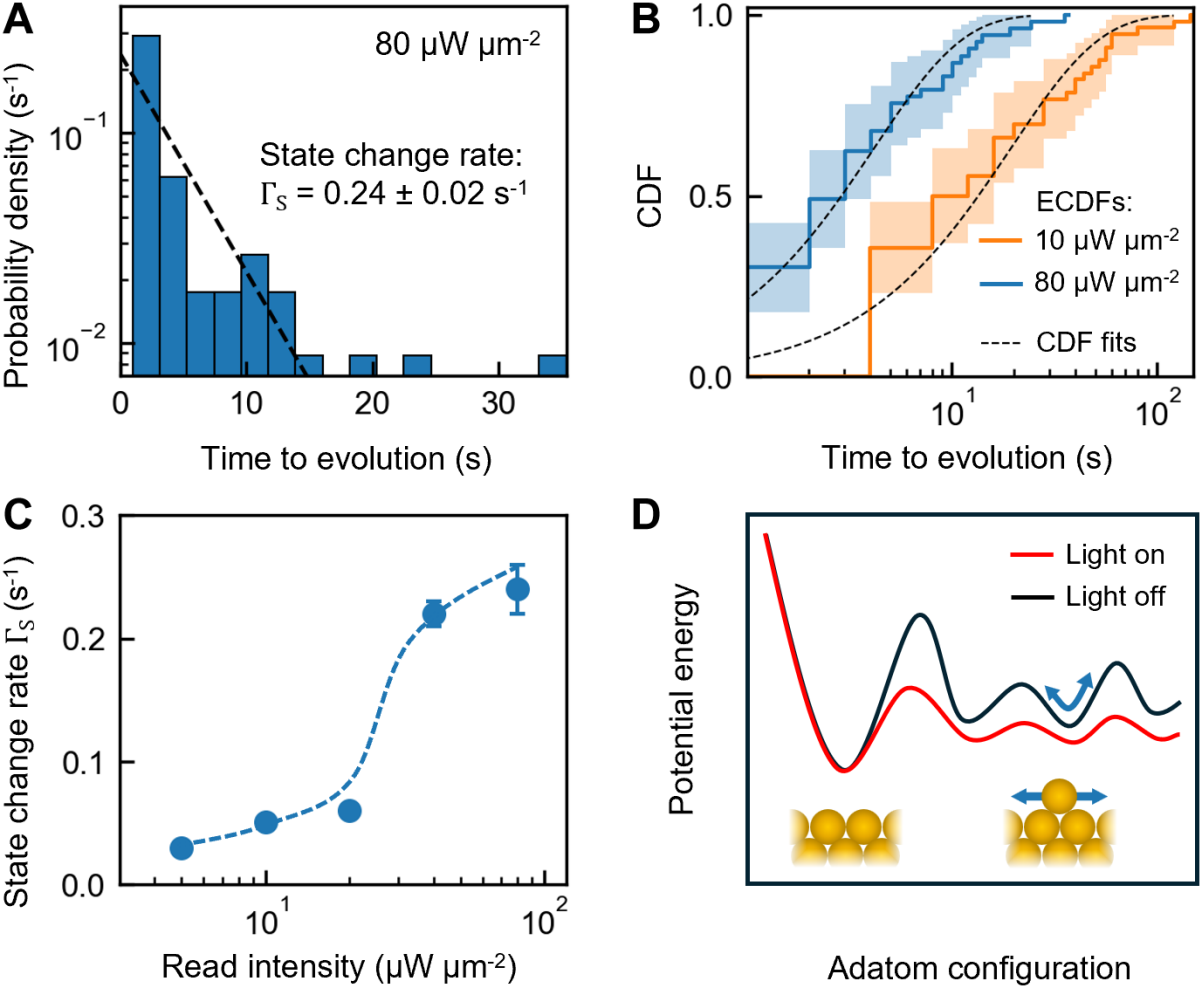
Picocavity state dynamics. (**A**) Example histogram of picocavity state evolution times, with exponential probability distribution (dashed line, $f_{S}=\Gamma_{S}e^{-\Gamma_{S}t}$ ) derived from fitting 80 μW μm^−2^ ECDF in (B). (**B**) Example ECDFs of picocavity state evolution time distributions along with exponential CDF fits ( $F_{S}=1-e^{-\Gamma_{S}t}$ ). Shaded regions denote 95% confidence intervals. (**C**) Picocavity state change rate $\Gamma_{S}$ dependence on laser intensity (line is guide to eye). Error bars are derived from the CDF least squares fits. (**D**) Schematic representation of energy landscape explored by the adatom, where formation, decay, and evolution barriers are all reduced under illumination.

It is therefore of interest to evaluate the stability of the initial read state after writing the picocavity (see Materials and Methods), and when it first evolves. The time distribution of when the initial picocavity state first evolves during the read stage appears exponential (Fig. 4A). Exponential CDFs are fitted to these ECDF (as above) to extract state change rates $\Gamma_{S}$ for each read intensity (Fig. 4B and fig. S4). The resulting power density dependence shows a distinct step in $\Gamma_{S}$ near 30 μW μm^−2^ (Fig. 4C). Such a step has already been observed for BPT picocavities at 10 K using a different metric for state changes (*24*).

This shows that reading at intensities <30 μW μm^−2^ is much more likely to preserve the initial state but does not guarantee it even at lowest intensities. Figure 3E and fig. S5 (I to M) show that spectral changes are prevalent even at ultralow illumination. This implies that ambient thermal energy alone may be enough for the adatom to explore its energy landscape in some configurations. Analogously to formation and decay barriers, the barriers for fluctuations seem also to be lowered under illumination through a similar mechanism (Eq. 1), making spectral wandering much more prominent (Fig. 4D). To prevent these thermally induced fluctuations after writing, molecules with intrinsically higher barriers to adatom fluctuation could be used, for instance, with different surface binding.

## DISCUSSION

**Fig. 5.**
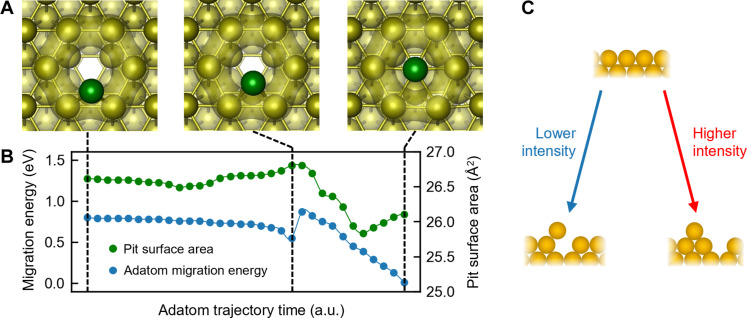
Adatom stability simulations. (**A**) Atomistic simulations showing configuration of adatom and pit (from which the adatom emerges and protrudes on the gold facet). Successive frames show adatom swinging over the lip of the pit, reentering it, and healing the surface. (**B**) Migration energy of the adatom and surface area of the pit in (A) versus simulated adatom trajectory time (a.u., arbitrary units). (**C**) Schematics showing side view of possible configurations of adatom structures corresponding to lower and higher write intensities: closely suspended versus separated states.

To model aspects of this fluctuation and decay landscape for picocavities, we use periodic DFT with nudged elastic band (NEB) ab initio molecular dynamics simulations to track the motion of Au adatoms on Au(111). The model and computational details follow earlier work (*57*) with details given in Methods and note S5. The model shows extraction energies of adatoms up out of the Au(111) surface of ~0.8 eV, leaving behind a pit vacancy to which the adatom stays weakly bound. We examine the diffusion of this adatom around the pit to better understand under what conditions there can be recombination of adatom and pit vacancy to clear the protrusion, corresponding to picocavity decay. We find that when the Au adatom moves back over its vacancy site, it is not always easily able to recombine. To recombine, the pit needs to briefly change shape (Fig. 5A and movies S1 and S2), introducing a ~0.3-eV energy barrier to recombination (Fig. 5B). Another ~0.2-eV barrier keeps the adatom from migrating away from the pit. With these barriers of order ~10 $\;k_{B}T$ locally restraining the adatom, we suggest that when they are closely suspended around the pit edge they will still recombine more easily than when the adatoms are fully separated from their pit and requiring additional migration along step edge or terrace sites to return. Adatoms are more prone to diffuse at higher irradiation intensities (Fig. 4C), so they can then move further away from their vacant pit, or other atoms can infiltrate the pit deforming or migrating the vacancy. Writing at lower intensity would create less stable closely suspended adatoms (Fig. 5C), thus explaining the decrease in read stability in this case (Fig. 2E). We note that the energy scales may change with coupled adatom-molecule and molecule-molecule dynamics in the experiments (*57*).

It would thus be highly desirable to include the light-induced energy barrier dependence into such models to further capture the dynamics displayed by this system. This would also open opportunities for developing more advanced control strategies, now that fast tuning of the irradiation (intensity, color, polarization) is possible since the current picocavity state is read out by SERS in real time. Work is in progress in this regard, but currently cannot yet be made rigorous without further advances, indicating the need for further theory developments in these single-atom extreme light-matter coupled systems.

To summarize, we demonstrate the deterministic formation or “writing” of adatoms in plasmonic nanogaps using bursts of mW μm^−2^ laser irradiation. By monitoring SERS spectra in real time and dynamically adjusting laser intensities, single picocavities can be deterministically created. The atomic-scale protrusions created provide additional sub–1 nm^3^ optical field localization termed picocavities. These picocavities exhibit intriguing photophysical and photochemical properties, including greatly boosting light-matter interactions to enable single-molecule SERS. We show that these deterministically created picocavities can be preserved for at least a week in the dark and probed with minimal risk of destruction using lower-power SERS. We find that the stability of written picocavities increases when using higher write intensities, which implies more extensive reconstruction or adatom movement, changing the energy landscape for adatom-vacancy recombination. Molecular dynamics simulations support this, as they find that rapid surface relaxation introduces additional energy barriers and can result in adatoms residing closely suspended next to their own vacancy pit when the write intensity is low.

These findings now allow the utilization of extreme molecule-plasmon interactions offered by picocavities. The controllable formation and prolonged stability of these atomic-scale features provide a route toward extended SERS monitoring of metal-molecule interactions (important for catalysis and molecular electronics), opto-mechanical devices, and single-molecule quantum optical applications. It also serves as a starting point for optically controlled atomic-scale memory and electronic devices. While extended stability is demonstrated, ambient thermal motion of the adatom-molecule is observed even in dark conditions as evidenced by SERS fluctuations, with many adatoms decaying or migrating. It would be interesting to further explore how alternative SAM molecules and metals can be used to stabilize the adatom under ambient conditions.

Finally, our findings suggest that these ideas can be generalized to all plasmonic hotspots, including other gold nanogap constructs beyond the NPoM structures demonstrated here such as bowtie antennas, dimers, aggregates, and in TERS applications. These findings are expected to also translate to different metals, although formation rates and the obtainable architectures will vary notably.

## MATERIALS AND METHODS

### Sample preparation

All chemicals were purchased from Sigma-Aldrich and used as received, unless stated otherwise. Template stripping was used to create an atomically smooth gold substrate (*22*, *23*, *28*). To create a BPT SAM, the substrate was immersed in 1 mM BPT in anhydrous ethanol for 18 hours. The substrate with SAM coating was washed with ethanol and dried under nitrogen flow. Standard aqueous 80-nm AuNPs with previously reported morphology (*41*) were purchased from BBI Solutions (*58*). Free-floating citrate is removed from the AuNP solution by centrifuging at 4500 relative centrifugal force (rcf) for 5 min and replacing the clear supernatant twice with deionized water. AuNPs were deposited on the gold substrate for 10 s by drop-casting 85 μl of the solution and mixing with 0.1 M aqueous NaNO_3_ in a 15:1 volume ratio. The sample was rinsed with deionized water and dried under nitrogen.

### SERS measurements

All Raman spectra were collected using a modified BX51 microscope coupled to a spectrometer. The sample position was controlled by a Prior Scientific motorized stage. Dark field images were recorded with a Lumenera Infinity2 charge-coupled device (CCD) camera. These were used to automatically center and focus on individual NPoMs. Independent 633- and 785-nm single-frequency diode lasers were used as Raman excitation sources. Laser powers on the sample were dynamically controlled by an AOM at either laser wavelength. The AOMs were calibrated by measuring the power on the sample with a Thorlabs PM16-121 power meter. The lasers were focused on a diffraction-limited spot of size ~1 μm. Light was coupled onto and from the sample via a dichroic beamsplitter and an Olympus MPLFLN100XBD NA (numerical aperture) 0.9 objective. Elastically scattered laser light was removed using two Iridian 632.8-16 NNF (633 nm) or two Iridian 785-20 NNF (785 nm) notch filters. Raman scattered light was spectrally resolved by a HORIBA Triax 320 spectrometer and imaged onto an Andor Newton EMCCD.

### Manual analysis

All picocavity write-read datasets are manually filtered to exclude datasets: (i) with notable stage drift, (ii) where check spectra are anomalous, (iii) where the first read spectrum is notably dissimilar to the final write spectrum or does not contain a picocavity, (iv) where the write triggers on something that is not a picocavity, e.g., cosmic ray or a broadband transient feature known as a flare (*28*, *54*, *59*), (v) that show very extensive persistent broadband features that mask picocavities after writing, attributed to photochemical changes. Lower and upper limit read lifetimes are extracted manually by measuring the lifetime of a picocavity from the read start. Correlations between peak movements, switching, and appearance/disappearance (*8*, *51*) are used to judge if spectra belong to the same picocavity and assign lower and upper limit lifetimes (see note S1). The lifetime data are converted into the proportion of picocavities that survive until the end of the read scan (200 s), yielding lower and upper limit lifetimes. Only the upper limit was used for Fig. 2E. The time to evolution is extracted as the time from start of a read scan before a first significant spectral change of the written picocavity is observed, either as a marked change in relative picocavity peak intensities or a peak shift of at least 0.5 nm.

### Atomistic simulations

The gold model was described using periodic plane wave DFT in the VASP code (*60*) with the generalized gradient approximation of Perdew-Burke-Ernzerhof (GGA-PBE) functional (*61*), projector augmented wave (PAW) (*62*) pseudopotentials with a plane wave cutoff of 400 eV, and Brillouin zone Γ-point sampling. We used a three-layer 192-atom slab for Au(111) with 30-Å vacuum spacing. Further details of the periodic surface models and DFT methods are in (*57*).

## Supplementary Materials

### The PDF file includes:

Figs. S1 to S5 Notes S1 to S5 Tables S1 and S2 Legends for movies S1 and S2 References
