# Supplementary material for: Optical control of single-atom dynamics in plasmonic nanogaps
Source: Sci Adv. 2025 Jul 18;11(29):eadx3216. doi: 10.1126/sciadv.adx3216 (PMC12273785; doi:10.1126/sciadv.adx3216)
Supplement: Supplementary file 1 — Figs. S1 to S5 Notes S1 to S5 Tables S1 and S2 Legends for movies S1 and S2 References [file sciadv.adx3216_sm.pdf]

Science Advances
AAAS

Supplementary Materials for

Optical control of single-atom dynamics in plasmonic nanogaps

Paul Kerner *et al.*

Corresponding author: Jeremy J. Baumberg, jjb12@cam.ac.uk; Bart de Nijs, bd355@cam.ac.uk

Sci. Adv. **11**, eadx3216 (2025)
DOI: 10.1126/sciadv.adx3216

The PDF file includes:

Figs. S1 to S5
Notes S1 to S5
Tables S1 and S2
Legends for movies S1 and S2
References

Other Supplementary Material for this manuscript includes the following:

Movies S1 and S2

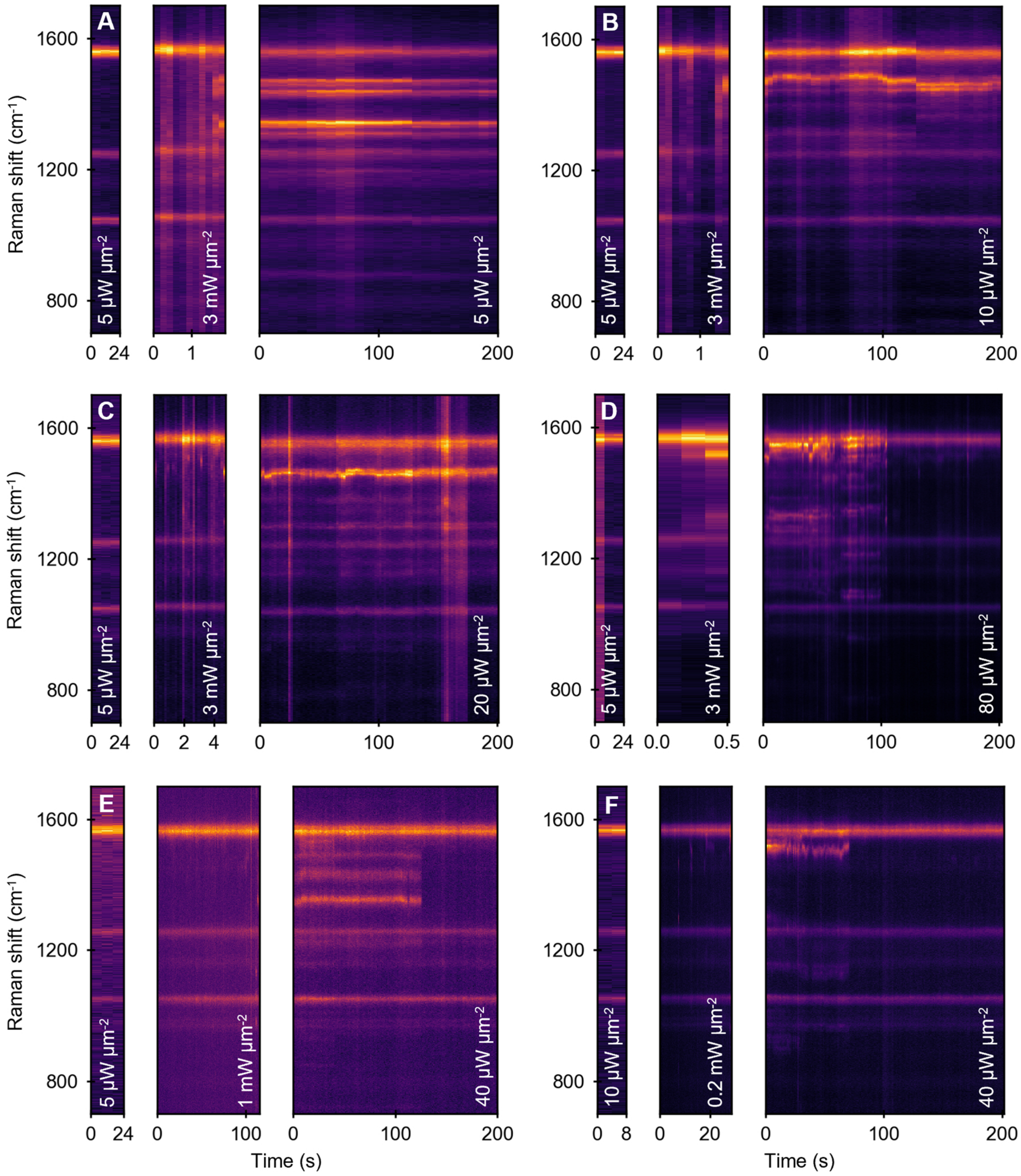

Figure S1. Further picocavity write-read scans. Picocavity write-read time-series SERS scans, where in each subfigure (A-F) the 1st panel corresponds to the low-power "*check*" stage, 2nd panel is high power "*write*" stage, and last panel is lower power "*read*" stage. Each panel is normalised separately to its maximum value. (**A**) *Check* at 5 µW µm^{-2}, 8 s exposure per spectrum; *write* at 3 mW µm^{-2}, 0.17 s irradiation per spectrum; *read* at 5 µW µm^{-2}, 8 s exposure per spectrum. (**B**) *Check* at 5 µW µm^{-2}, 8 s exposure per spectrum; *write* at 3 mW µm^{-2}, 0.17 s irradiation per spectrum; *read* at 10 µW µm^{-2}, 4 s exposure per spectrum. (**C**) *Check* at 5 µW µm^{-2}, 8 s exposure per spectrum; *write* at 3 mW µm^{-2}, 0.17 s irradiation per spectrum; *read* at 20 µW µm^{-2}, 2 s exposure per spectrum. (**D**) *Check* at 5 µW µm^{-2}, 8 s exposure per spectrum; *write* at 3 mW µm^{-2}, 0.17 s irradiation per spectrum; *read* at 80 µW µm^{-2}, 1 s exposure per spectrum. (**E**) *Check* at 5 µW µm^{-2}, 8 s exposure per spectrum; *write* at 1 mW µm^{-2}, 0.17 s irradiation per spectrum; *read* at 40 µW µm^{-2}, 1 s exposure per spectrum. (**F**) *Check* at 10 µW µm^{-2}, 4 s exposure per spectrum; *write* at 0.2 mW µm^{-2}, 0.2 s irradiation per spectrum; *read* at 40 µW µm^{-2}, 1 s exposure per spectrum.

Supplementary Note S1. Read lifetime extraction

After filtering, read spectra are manually processed to extract the lifetime of the picocavity created in the preceding writing stage. The lifetime is determined from the number of spectra that the starting picocavity stays alive for. However, as picocavity peaks often shift and switch in intensity and frequency (*24*, *51*), it is often not clear when and if a picocavity decays, switches to a new state, or a new picocavity is formed.

Besides this, multi-second long periods of significantly diminished or completely absent signal are observed, after which picocavity peaks return to similar positions and intensities. These dark periods would normally be classed as picocavity decay by automated analysis (*28*) but could imply the existence of low or non-scattering picocavity states. Even if peaks in different positions are observed after a dark period, we cannot rule out that they belong to the same picocavity which may switch to a third state after the dark state. Dark states are further confirmed by the fact that read scans are mostly performed at power densities where new picocavity formation is unlikely.

A set of guidelines is developed to assign the likelihood of read spectra still containing the initial picocavity. The guidelines rely on the fact that peaks of a single picocavity tend to shift and switch in time in a correlated or anti-correlated manner (*8*, *51*). If multiple picocavities are observed from the start (multiple sets of peaks that are not intercorrelated), only the brighter one that triggered the write condition are tracked and prioritised.

Spectra are classed **highly likely** to contain the starting picocavity if the picocavity peaks in the preceding spectra only:

- stay the same and do not significantly change in intensity or position (Fig. S2A).
- gradually shift in position or change in intensity (Fig. S2B).
- switch to new positions abruptly, i.e. one peak disappears, and a new one appears in a different part of the subsequent spectrum (Fig. S2C).
- switch and shift in an (anti)correlated manner for multiple peaks, i.e. if one peak switches or moves, so do the others (Fig. S2, B-C).
- reappear at similar intensities and positions after dark periods (Fig. S2D).

Spectra are classed **less likely** to contain the starting picocavity if the picocavity peaks in the preceding spectra additionally:

- strongly dim in intensity, leaving behind only faint peaks in similar positions (Fig. S2E). The faint peaks could belong to a different picocavity already present, masked by the brighter peaks. Alternatively, they could belong to a dimmer state of the previously bright picocavity.
- disappear, but other fainter peaks remain unchanged and keep going (Fig. S2F). The unchanged peaks might belong to a different picocavity present from the start. Alternatively, they could belong to the same initial picocavity, but the state change could preserve some peaks and/or experience changes in intensity/position below the detection limit. If the unchanged peaks are brighter than the disappearing peaks, we keep tracking the brighter peaks, assigning them high likelihood to belong to the initial picocavity.
- reappear at different positions or intensities after dark periods, but still have similar fluctuations and/or intensities (Fig. S2G).

Spectra are classed **unlikely** to contain the starting picocavity if the picocavity peaks in the preceding spectra:

- all disappear or strongly diminish to near the detection limit and do not subsequently brighten again (Fig. S2H).

- appear without a correlated change in other present peaks (Fig. S2I). This signifies a new picocavity forming and such new peaks are ignored.
- all disappear and leave behind only broadband features, attributable to plasmonic flares (*28*, *54*, *59*) or photochemical changes (Fig. S2J).

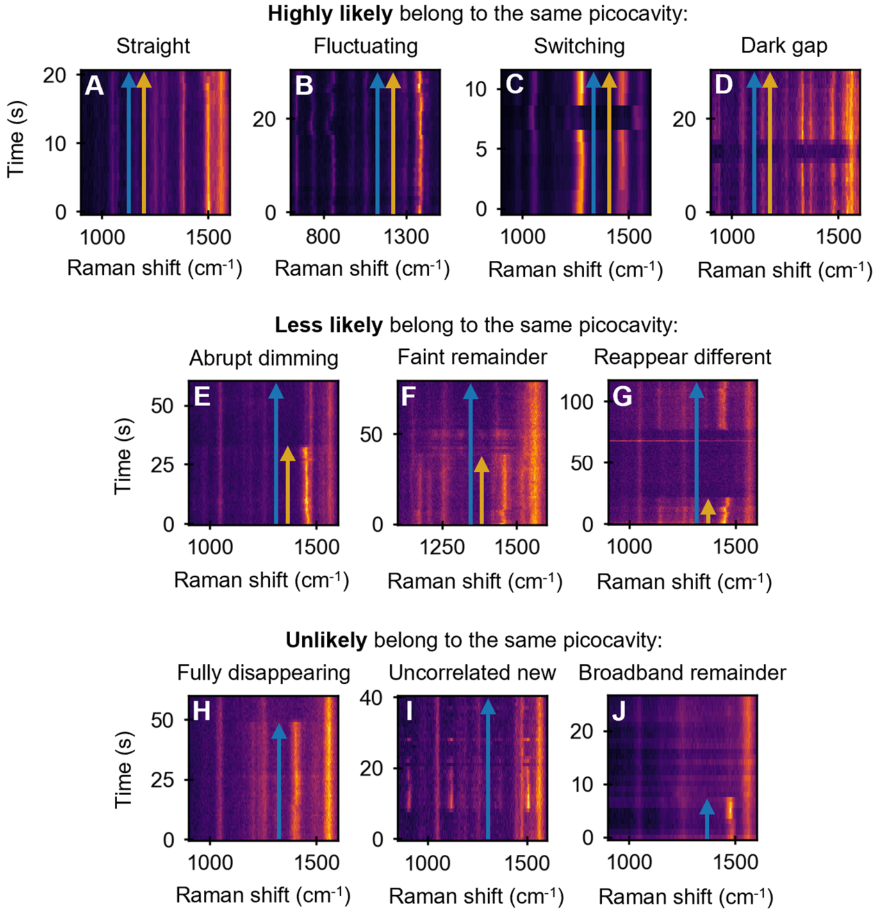

Figure S2. Picocavity read spectra analysis. (**A-J**) Examples of observed spectral behaviour types in read spectra and their assignments of likelihood of subsequent spectra to still contain the initially-written picocavity (see Supplementary Note S1 for detailed descriptions of these behaviours). Arrows track all spectra that are **highly likely** (blue) and **less likely** (yellow) to retain the initial picocavity, indicating the **upper** and **lower limits** of read lifetimes.

Two picocavity lifetimes are extracted for every read timescan. The time from the start until the last **highly likely** spectrum is the **lower limit** read lifetime. The time from the start until the first **unlikely** spectrum (including any **less likely** spectra) is the **upper limit** read lifetime.

A significant proportion of picocavities are found to survive until the end of the scan. Fitting a distribution of lifetimes is thus not possible and the percentage of picocavities that survive until the end of the scan (200 s) known as the survival percentage, is used as a figure of merit. The lower and upper limit lifetimes provide respectively the lower and upper limit survival percentages. Uncertainty limits $\acute{z}_{/}$ are calculated from the binomial distribution variance

$$\acute{z}_{/}^{2} = {}^{x}\ \sim(1 - \sim-, \qquad \text{(S1)}$$

where ^{x} is the total number of measurements at one power density and $\sim$ is the probability that the picocavity survives until the end of the scan. $\sim$ is estimated as the survival percentage for the purposes of $\acute{z}_{/}$. The uncertainty in survival percentage is $100\% \times \acute{z}_{/} /$.

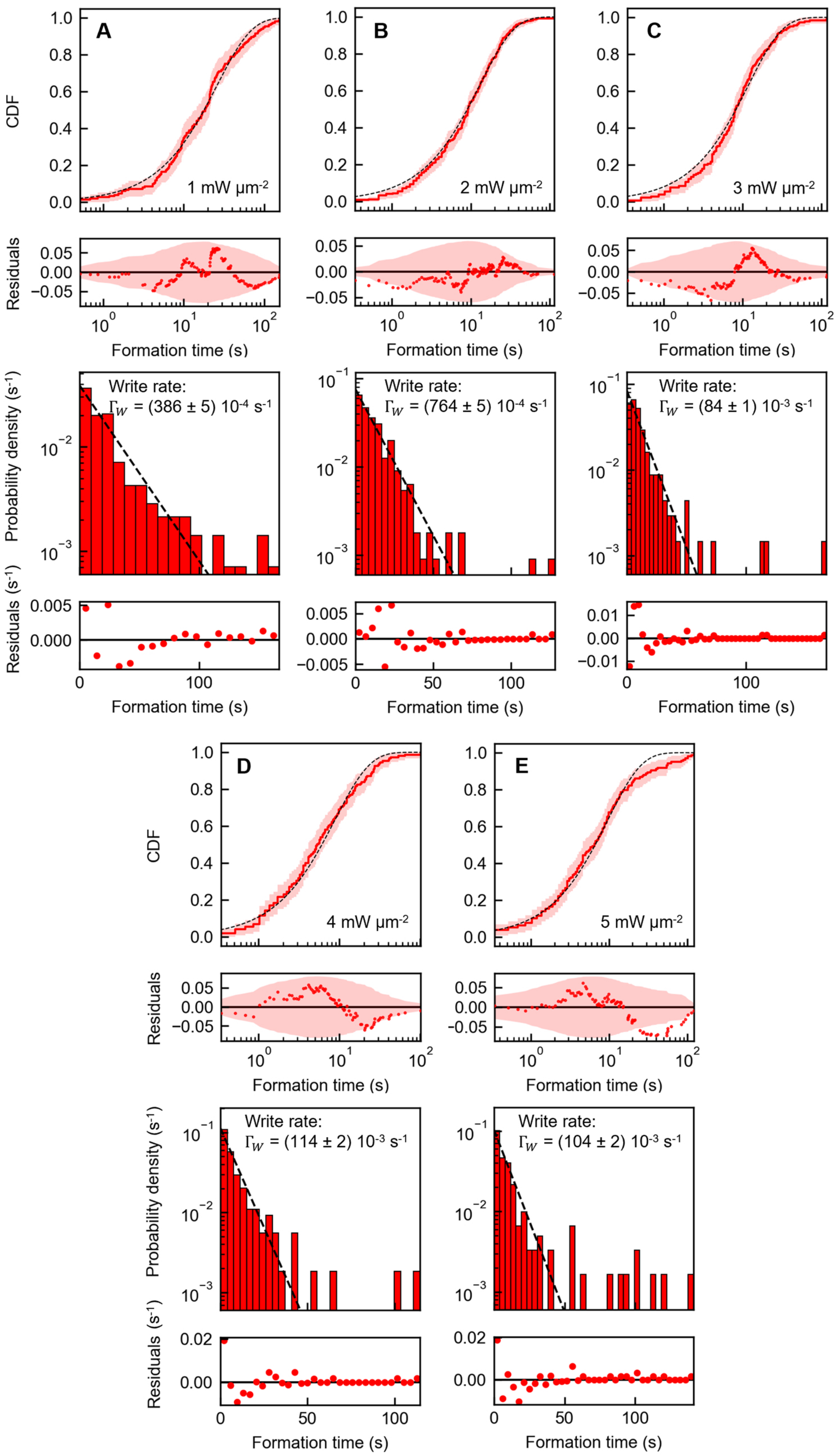

Figure S3. Picocavity write statistics. (**A**-**E**) Upper panels: empirical cumulative density distribution functions (ECDF) of picocavity write time distributions at 1, 2, 3, 4, and 5 mW μm^{-2} write intensities, along with exponential CDF fits ($1 - e^{-\Gamma_W t}$) (dashed), with fit residuals. Shaded regions denote 95% confidence intervals. Lower panels: histograms of picocavity write times, with exponential probability distribution functions (dashed line; $\Gamma_W e^{-\Gamma_W t}$) derived from the CDF fits above.

Supplementary Note S2. Write rate statistics

Picocavity write time distributions at write intensities of 1, 2, 3, 4 and 5 mW µm^{-2} are obtained by writing a picocavity into a number of NPoMs at each intensity. 152 picocavities are written at 1 mW µm^{-2}, 269 at 2 mW µm^{-2}, 186 at 3 mW µm^{-2}, 148 at 4 mW µm^{-2}, 158 at 5 mW µm^{-2}. The write times are directly extracted from the number of 170 ms irradiation cycles needed to create the picocavities.

The obtained write time distributions are converted to empirical cumulative distribution functions (ECDF) (*63*),

$$\hat{F}_n(t) = \frac{\text{number of elements in sample} \leq t}{[illegible]}. \quad \text{(S2)}$$

The ECDF approaches the cumulative distribution function (CDF) of the distribution as [illegible] grows. The exponential CDF-s,

$$F_{\mathrm{A}}(t) = 1 - [illegible] \quad \text{(S3)}$$

are least-squares fits to the write time ECDF-s, with write rate Γ_{A} as the fit parameter (Fig. S3, upper panels). 95% confidence intervals are calculated for the ECDF-s (*64*) and used for the least-squares fits. Probability density histograms of the write time distributions are visually compared to the derived exponential probability density functions,

$$[illegible]_{\mathrm{A}}(t) = \Gamma_{\mathrm{A}} [illegible] \quad \text{(S4)}$$

to ensure the fits are reasonable (Fig. S3, lower panels). The dependence of write rate Γ_{A} on write intensity $[illegible]_{\mathrm{A}}$ is shown in the main text Fig. 2C.

Supplementary Note S3. Read intensity dependence statistics

Picocavity read data is collected for read intensities of 5, 10, 20, 40 and 80 µW µm^{-2} for a number of picocavities written at 3 mW µm^{-2} in separate NPoMs. The extracted statistics for each intensity (Table S1) are percentages of picocavities that survive for 200 s of read intensity (lower and upper limits $[illegible]$ and $[illegible]$ as defined in Supplementary Note S1) (main text Fig. 2D) and the state change rate Γ_1 (main text Fig. 4C).

Table S1. Read statistics for picocavities written at 3 mW µm^{-2} laser intensity and a range of read intensities. $[illegible]_2$ is read intensity. [illegible] is the total number of successfully written picocavities with that read intensity, [illegible] is the percentage of NPoMs where a picocavity was successfully written (see Methods for exclusion criteria). [illegible] and [illegible] are lower and upper limits for the percentage of picocavities that survive for 200 s (as defined in Supplementary Note S1) at indicated read intensity. Γ_1 is the extracted picocavity state change rate (Fig. S4). Errors in [illegible], [illegible] are calculated from the binomial distribution variances (Eq. S1). Errors in Γ_1 are obtained from the least squares fits.

[illegible] [illegible]	[illegible] ([illegible]) [%]	[illegible] [%]	[illegible] [%]	[illegible] [[illegible]]
5	61 (53%)	74 ± 6	92 ± 4	0.030 ± 0.001
10	56 (65%)	70 ± 6	84 ± 5	0.051 ± 0.004
20	52 (56%)	55 ± 7	76 ± 6	0.060 ± 0.004
40	56 (63%)	36 ± 6	71 ± 6	0.22 ± 0.01
80	53 (57%)	26 ± 6	66 ± 7	0.24 ± 0.02

As outlined in Methods, the picocavity time until evolution ($[illegible]_1$) is defined as the time from the start of a read scan until picocavity lines significantly change in intensity or shift by at least 0.5 nm. Similar to Supplementary Note S2, ECDF-s for $[illegible]_1$ distributions for each read intensity $[illegible]_2$ are used to fit exponential CDF-s,

$$F_1(t) = 1 - [illegible] \quad \text{(S5)}$$

to extract Γ_1 (Fig. S4). The corresponding exponential probability density functions are

$$[illegible]_1(t) = \Gamma_1 [illegible]. \quad \text{(S6)}$$

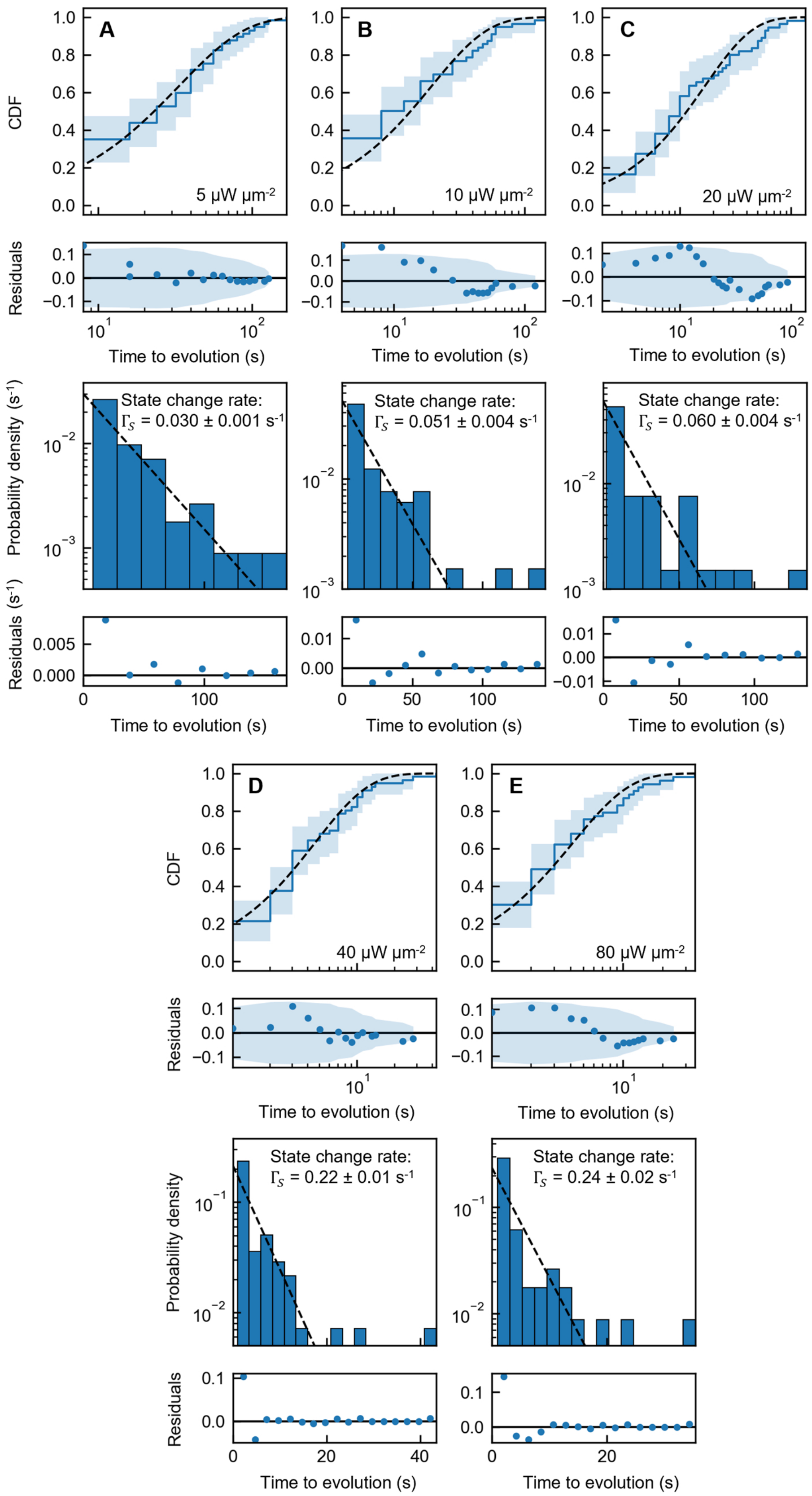

Figure S4. Picocavity state change statistics. (**A**-**E**) Upper panels: empirical cumulative density distribution functions (ECDF) of picocavity evolution time distributions at 5, 10, 20, 40, 80 µW µm^{-2} read intensities, along with exponential CDF fits (1 − [illegible]) (dashed), with fit residuals. Shaded regions denote 95% confidence intervals. Lower panels: histograms of picocavity evolution times, with exponential probability distribution functions (dashed line; Γ_1 [illegible]) derived from the CDF fits above.

Supplementary Note S4. Read dependence on write intensity

Separate write-read experiments are also performed with write intensities of 0.2, 0.5, 1, 3 and 5 mW µm^{-2} and 40 µW µm^{-2} read intensity to determine the read survival dependence on write intensity. Upper limit read survival percentages $._{0}$ are extracted from the read scans as discussed in Supplementary Note S1 (Table S2).

Table S2. Read statistics for picocavities written at a range of intensities and read at 40 µW µm^{-2}. $-_{A}$ is write intensity.^{x} $_{3\ 456678}$ is the total number of successfully written picocavities at that intensity, $9_{3\ 456678}$ is the percentage of NPoMs where a picocavity was successfully written (see Methods for exclusion criteria). $._{0}$ is the upper limit for the percentage of picocavities that survive for 200 s (as defined in Supplementary Note S1) at 40 µW µm^{-2} read intensity after being written at indicated intensity. Errors in $._{0}$ are calculated from the binomial distribution variances (Eq. S1).

$:_{Q}$ [? > =? ⊕@]	$B_{C\ DEIFGH}$ (; $_{C\ DEIFGH}$ [%]	I_{K} [%]
0.2	40 (49%)	24 ± 7
0.5	42 (47%)	40 ± 8
1	59 (50%)	58 ± 6
3	75 (66%)	68 ± 5
5	96 (77%)	79 ± 4

Supplementary Note S5. Atomistic simulations of adatom recombination

Gold adatom recombination with its pit vacancy on a gold (111) surface was simulated as detailed in Methods. The adatom follows the re-entrance trajectory starting on the lip of the pit, simulated across 32 frames (Fig. 5A-B). Top and side view animations of the trajectory are supplied with Supplementary Movies S1 and S2. The Nudged Elastic Band (NEB) method was used with >30 images to fully explore the detailed recombination trajectory around the pit with all atoms in the top two surface layers including the adatom relaxed unconstrained until the forces on each atom were <3 meV/Å, and the bottom Au layer held fixed to approximate coupling to bulk metal.

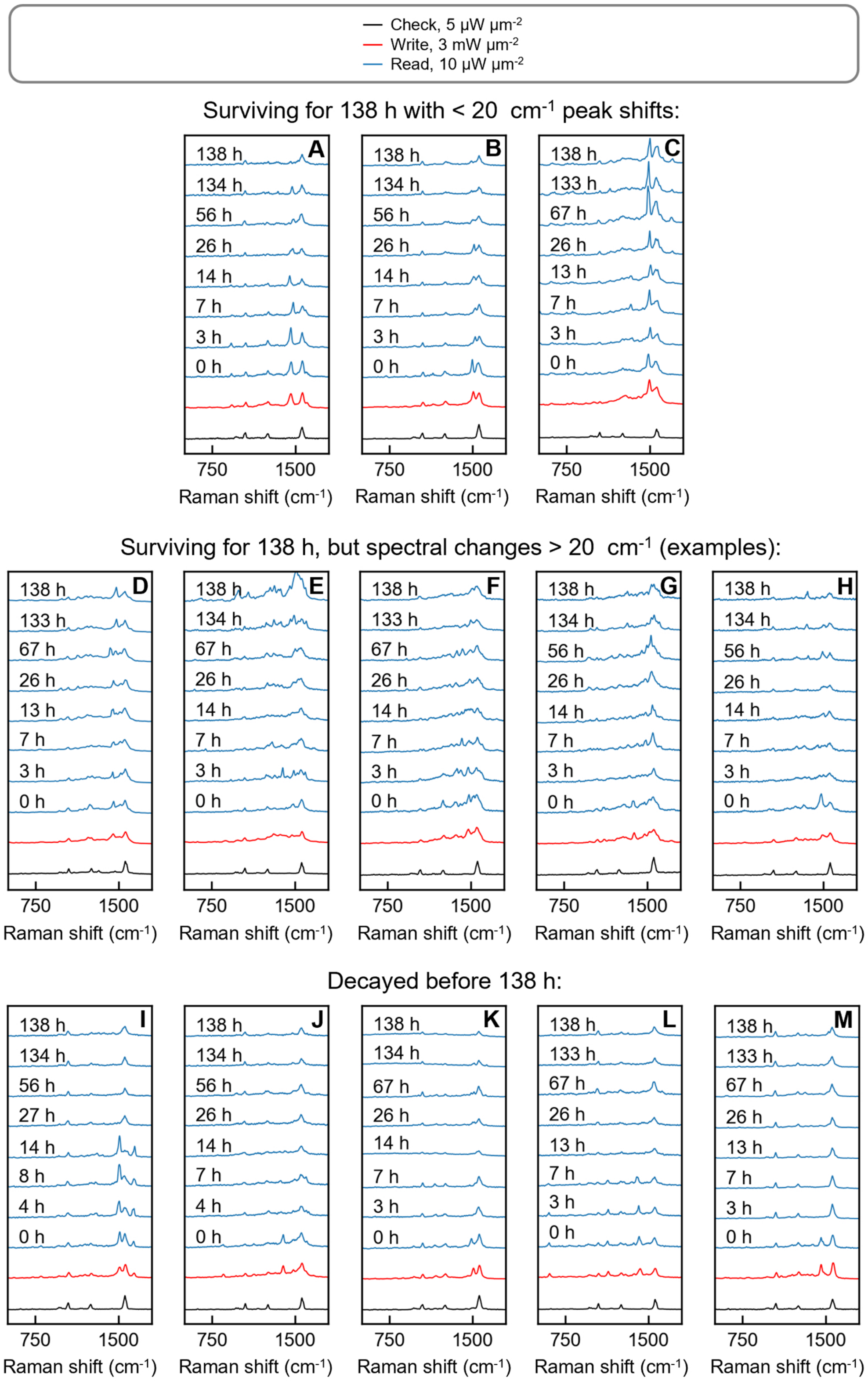

Figure S5. Long-term storage in darkness. 633 nm excitation SERS spectra resulting from write-read experiments on 32 picocavities over the course of a week, with increasing periods of dark storage between 10 μW μm^{-2} read spectra. Only the final write spectra at 3 mW μm^{-2} (red) are shown. All spectra in are normalised to the 1050 cm^{-1} nanocavity peak and smoothed with a 3rd order 11 pt Savitzky-Golay filter. (**A**-**C**) 3 picocavities that survived for the whole duration of the measurement (138 h) without significant spectral changes > 20 cm^{-1} or peak (dis)appearances. (**D**-**H**) Examples of the 24 picocavities that showed significant spectral shifts > 20 cm^{-1} or peaks appearing/disappearing over the course of the measurement (**I**-**M**) 5 picocavities that decayed over the course of the measurement.

Supplementary Movie S1. Adatom recombination top view. Top view animation of the simulated adatom trajectory shown in Fig. 5, A-B.

Supplementary Movie S2. Adatom recombination side view. Side view animation of the simulated adatom trajectory shown in Fig. 5, A-B.

REFERENCES AND NOTES

1. N. Xin, J. Guan, C. Zhou, X. Chen, C. Gu, Y. Li, M. A. Ratner, A. Nitzan, J. F. Stoddart, X. Guo, Concepts in the design and engineering of single-molecule electronic devices. *Nat. Rev. Phys.* **1**, 211–230 (2019).

2. H. Song, M. A. Reed, T. Lee, Single molecule electronic devices. *Adv. Mater.* **23**, 1583–1608 (2011).

3. L. Meng, N. Xin, C. Hu, H. A. Sabea, M. Zhang, H. Jiang, Y. Ji, C. Jia, Z. Yan, Q. Zhang, L. Gu, X. He, P. Selvanathan, L. Norel, S. Rigaut, H. Guo, S. Meng, X. Guo, Dual-gated single-molecule field-effect transistors beyond Moore's law. *Nat. Commun.* **13**, 1410 (2022).

4. M. Fuechsle, J. A. Miwa, S. Mahapatra, H. Ryu, S. Lee, O. Warschkow, L. C. L. Hollenberg, G. Klimeck, M. Y. Simmons, A single-atom transistor. *Nat. Nanotech.* **7**, 242–246 (2012).

5. C. Schirm, M. Matt, F. Pauly, J. C. Cuevas, P. Nielaba, E. Scheer, A current-driven single-atom memory. *Nat. Nanotech.* **8**, 645–648 (2013).

6. H. P. Specht, C. Nölleke, A. Reiserer, M. Uphoff, E. Figueroa, S. Ritter, G. Rempe, A single-atom quantum memory. *Nature* **473**, 190–193 (2011).

7. L. Zhou, D. F. Swearer, C. Zhang, H. Robatjazi, H. Zhao, L. Henderson, L. Dong, P. Christopher, E. A. Carter, P. Nordlander, N. J. Halas, Quantifying hot carrier and thermal contributions in plasmonic photocatalysis. *Science* **362**, 69–72 (2018).

8. A. Stefancu, J. Aizpurua, I. Alessandri, I. Bald, J. J. Baumberg, L. V. Besteiro, P. Christopher, M. Correa-Duarte, B. de Nijs, A. Demetriadou, R. R. Frontiera, T. Fukushima, N. J. Halas, P. K. Jain, Z. H. Kim, D. Kurouski, H. Lange, J.-F. Li, L. M. Liz-Marzán, I. T. Lucas, A. J. Meixner, K. Murakoshi, P. Nordlander, W. J. Peveler, R. Quesada-Cabrera, E. Ringe, G. C. Schatz, S. Schlücker, Z. D. Schultz, E. X. Tan, Z.-Q. Tian, L. Wang, B. M. Weckhuysen, W. Xie, X. Y. Ling, J. Zhang, Z. Zhao, R.-Y. Zhou, E. Cortés, Impact of surface enhanced raman spectroscopy in catalysis. *ACS Nano* **18**, 29337–29379 (2024).

9. A. Vojvodic, A. J. Medford, F. Studt, F. Abild-Pedersen, T. S. Khan, T. Bligaard, J. K. Nørskov, Exploring the limits: A low-pressure, low-temperature Haber–Bosch process. *Chem. Phys. Lett.* **598**, 108–112 (2014).

10. X.-F. Yang, A. Wang, B. Qiao, J. Li, J. Liu, T. Zhang, Single-atom catalysts: A new frontier in heterogeneous catalysis. *Acc. Chem. Res.* **46**, 1740–1748 (2013).

11. S. Qin, J. Will, H. Kim, N. Denisov, S. Carl, E. Spiecker, P. Schmuki, Single atoms in photocatalysis: Low loading is good enough! *ACS Energy Lett.* **8**, 1209–1214 (2023).

12. J. Zhou, J. Guo, A. M. Mebel, G. Ghimire, F. Liang, S. Chang, J. He, Probing the intermediates of catalyzed dehydration reactions of primary amide to nitrile in plasmonic junctions. *ACS Catal.* **12**, 7737–7747 (2022).

13. Y. Wang, F. Chu, J. Zeng, Q. Wang, T. Naren, Y. Li, Y. Cheng, Y. Lei, F. Wu, Single atom catalysts for fuel cells and rechargeable batteries: Principles, advances, and opportunities. *ACS Nano* **15**, 210–239 (2021).

14. J. Shin, Y. J. Lee, A. Jan, S. M. Choi, M. Y. Park, S. Choi, J. Y. Hwang, S. Hong, S. G. Park, H. J. Chang, M. K. Cho, J. P. Singh, K. H. Chae, S. Yang, H.-I. Ji, H. Kim, J.-W. Son, J.-H. Lee, B.-K. Kim, H.-W. Lee, J. Hong, Y. J. Lee, K. J. Yoon, Highly active and thermally stable single-atom catalysts for high-temperature electrochemical devices. *Energ. Environ. Sci.* **13**, 4903–4920 (2020).

15. J.-N. Longchamp, S. Rauschenbach, S. Abb, C. Escher, T. Latychevskaia, K. Kern, H.-W. Fink, Imaging proteins at the single-molecule level. *Proc. Natl. Acad. Sci. U.S.A.* **114**, 1474–1479 (2017).

16. S. Van Aert, K. J. Batenburg, M. D. Rossell, R. Erni, G. Van Tendeloo, Three-dimensional atomic imaging of crystalline nanoparticles. *Nature* **470**, 374–377 (2011).

17. C. Chen, P. Chu, C. A. Bobisch, D. L. Mills, W. Ho, Viewing the interior of a single molecule: Vibronically resolved photon imaging at submolecular resolution. *Phys. Rev. Lett.* **105**, 217402 (2010).

18. L. Gross, F. Mohn, N. Moll, P. Liljeroth, G. Meyer, The chemical structure of a molecule resolved by atomic force microscopy. *Science* **325**, 1110–1114 (2009).

19. M. Richard-Lacroix, V. Deckert, Direct molecular-level near-field plasmon and temperature assessment in a single plasmonic hotspot. *Light Sci. Appl.* **9**, 35 (2020).

20. A. Rosławska, P. Merino, A. Grewal, C. C. Leon, K. Kuhnke, K. Kern, Atomic-scale structural fluctuations of a plasmonic cavity. *Nano Lett.* **21**, 7221–7227 (2021).

21. S. Liu, F. P. Bonafe, H. Appel, A. Rubio, M. Wolf, T. Kumagai, Inelastic Light scattering in the vicinity of a single-atom quantum point contact in a plasmonic picocavity. *ACS Nano* **17**, 10172–10180 (2023).

22. F. Benz, M. K. Schmidt, A. Dreismann, R. Chikkaraddy, Y. Zhang, A. Demetriadou, C. Carnegie, H. Ohadi, B. De Nijs, R. Esteban, J. Aizpurua, J. J. Baumberg, Single-molecule optomechanics in "picocavities". *Science* **354**, 726–729 (2016).

23. C. Carnegie, J. Griffiths, B. De Nijs, C. Readman, R. Chikkaraddy, W. M. Deacon, Y. Zhang, I. Szabó, E. Rosta, J. Aizpurua, J. J. Baumberg, Room-temperature optical picocavities below 1 nm ^{3} accessing single-atom geometries. *J. Phys. Chem. Lett.* **9**, 7146–7151 (2018).

24. J. Griffiths, T. Földes, B. De Nijs, R. Chikkaraddy, D. Wright, W. M. Deacon, D. Berta, C. Readman, D.-B. Grys, E. Rosta, J. J. Baumberg, Resolving sub-angstrom ambient motion through reconstruction from vibrational spectra. *Nat. Commun.* **12**, 6759 (2021).

25. J. Griffiths, B. De Nijs, R. Chikkaraddy, J. J. Baumberg, Locating single-atom optical picocavities using wavelength-multiplexed raman scattering. *ACS Photonics* **8**, 2868–2875 (2021).

26. S. Schlücker, Surface-enhanced raman spectroscopy: Concepts and chemical applications. *Angew. Chem. Int. Ed.* **53**, 4756–4795 (2014).

27. J. J. Baumberg, Picocavities: A primer. *Nano Lett.* **22**, 5859–5865 (2022).

28. Q. Lin, S. Hu, T. Földes, J. Huang, D. Wright, J. Griffiths, E. Elliott, B. De Nijs, E. Rosta, J. J. Baumberg, Optical suppression of energy barriers in single molecule-metal binding. *Sci. Adv.* **8**, eabp9285 (2022).

29. S. Trautmann, J. Aizpurua, I. Götz, A. Undisz, J. Dellith, H. Schneidewind, M. Rettenmayr, V. Deckert, A classical description of subnanometer resolution by atomic features in metallic structures. *Nanoscale* **9**, 391–401 (2017).

30. M. Urbieta, M. Barbry, Y. Zhang, P. Koval, D. Sánchez-Portal, N. Zabala, J. Aizpurua, Atomic-scale lightning rod effect in plasmonic picocavities: A classical view to a quantum effect. *ACS Nano* **12**, 585–595 (2018).

31. M. Barbry, P. Koval, F. Marchesin, R. Esteban, A. G. Borisov, J. Aizpurua, D. Sánchez-Portal, Atomistic near-field nanoplasmonics: Reaching atomic-scale resolution in nanooptics. *Nano Lett.* **15**, 3410–3419 (2015).

32. R. Chikkaraddy, V. A. Turek, Q. Lin, J. Griffiths, B. De Nijs, U. F. Keyser, J. J. Baumberg, Dynamics of deterministically positioned single-bond surface-enhanced Raman scattering from DNA origami assembled in plasmonic nanogaps. *J. Raman Spectrosc.* **52**, 348–354 (2021).

33. R. Chikkaraddy, R. Arul, L. A. Jakob, J. J. Baumberg, Single-molecule mid-infrared spectroscopy and detection through vibrationally assisted luminescence. *Nat. Photon.* **17**, 865–871 (2023).

34. A. Boehmke Amoruso, R. A. Boto, E. Elliot, B. De Nijs, R. Esteban, T. Földes, F. Aguilar-Galindo, E. Rosta, J. Aizpurua, J. J. Baumberg, Uncovering low-frequency vibrations in surface-enhanced Raman of organic molecules. *Nat. Commun.* **15**, 6733 (2024).

35. J. Huang, D.-B. Grys, J. Griffiths, B. De Nijs, M. Kamp, Q. Lin, J. J. Baumberg, Tracking interfacial single-molecule pH and binding dynamics via vibrational spectroscopy. *Sci. Adv.* **7**, eabg1790 (2021).

36. J. Zhou, J. Guo, G. Ghimire, A. M. Mebel, S. Chang, J. He, Plasmon-mediated dehydrogenation of the aromatic methyl group and benzyl radical formation. *Chem. Sci.* **14**, 13951–13961 (2023).

37. J. J. Baumberg, J. Aizpurua, M. H. Mikkelsen, D. R. Smith, Extreme nanophotonics from ultrathin metallic gaps. *Nat. Mater.* **18**, 668–678 (2019).

38. C. Tserkezis, R. Esteban, D. O. Sigle, J. Mertens, L. O. Herrmann, J. J. Baumberg, J. Aizpurua, Hybridization of plasmonic antenna and cavity modes: Extreme optics of nanoparticle-on-mirror nanogaps. *Phys. Rev. A* **92**, 053811 (2015).

39. L. Tong, H. Xu, M. Käll, Nanogaps for SERS applications. *MRS Bull.* **39**, 163–168 (2014).

40. J. K. Daniels, G. Chumanov, Nanoparticle−mirror sandwich substrates for surface-enhanced raman scattering. *J. Phys. Chem. B* **109**, 17936–17942 (2005).

41. F. Benz, R. Chikkaraddy, A. Salmon, H. Ohadi, B. De Nijs, J. Mertens, C. Carnegie, R. W. Bowman, J. J. Baumberg, SERS of individual nanoparticles on a mirror: Size does matter, but so does shape. *J. Phys. Chem. Lett.* **7**, 2264–2269 (2016).

42. M. K. Kinnan, G. Chumanov, Surface enhanced raman scattering from silver nanoparticle arrays on silver mirror films: Plasmon-induced electronic coupling as the enhancement mechanism. *J. Phys. Chem. C* **111**, 18010–18017 (2007).

43. M. M. Schmidt, E. A. Farley, M. A. Engevik, T. N. Adelsman, A. Tuckmantel Bido, N. D. Lemke, A. G. Brolo, N. C. Lindquist, High-speed spectral characterization of single-molecule SERS fluctuations. *ACS Nano* **17**, 6675–6686 (2023).

44. N. C. Lindquist, A. G. Brolo, Ultra-high-speed dynamics in surface-enhanced raman scattering. *J. Phys. Chem. C* **125**, 7523–7532 (2021).

45. C. D. L. De Albuquerque, K. M. Hokanson, S. R. Thorud, R. G. Sobral-Filho, N. C. Lindquist, A. G. Brolo, Dynamic Imaging of multiple SERS hotspots on single nanoparticles. *ACS Photonics* **7**, 434–443 (2020).

46. H. Häkkinen, The gold–sulfur interface at the nanoscale. *Nat. Chem.* **4**, 443–455 (2012).

47. J. C. Love, L. A. Estroff, J. K. Kriebel, R. G. Nuzzo, G. M. Whitesides, Self-assembled monolayers of thiolates on metals as a form of nanotechnology. *Chem. Rev.* **105**, 1103–1170 (2005).

48. F. Benz, C. Tserkezis, L. O. Herrmann, B. De Nijs, A. Sanders, D. O. Sigle, L. Pukenas, S. D. Evans, J. Aizpurua, J. J. Baumberg, Nanooptics of molecular-shunted plasmonic nanojunctions. *Nano Lett.* **15**, 669–674 (2015).

49. M. Kamp, B. De Nijs, N. Kongsuwan, M. Saba, R. Chikkaraddy, C. A. Readman, W. M. Deacon, J. Griffiths, S. J. Barrow, O. S. Ojambati, D. Wright, J. Huang, O. Hess, O. A. Scherman, J. J. Baumberg, Cascaded nanooptics to probe microsecond atomic-scale phenomena. *Proc. Natl. Acad. Sci. U.S.A.* **117**, 14819–14826 (2020).

50. H.-H. Shin, G. J. Yeon, H.-K. Choi, S.-M. Park, K. S. Lee, Z. H. Kim, Frequency-domain proof of the existence of atomic-scale SERS hot-spots. *Nano Lett.* **18**, 262–271 (2018).

51. A. Poppe, J. Griffiths, S. Hu, J. J. Baumberg, M. Osadchy, S. Gibson, B. De Nijs, Mapping atomic-scale metal–molecule interactions: Salient feature extraction through autoencoding of vibrational spectroscopy data. *J. Phys. Chem. Lett.* **14**, 7603–7610 (2023).

52. Y. Zhang, Z. Dong, J. Aizpurua, Theoretical treatment of single-molecule scanning Raman picoscopy in strongly inhomogeneous near fields. *J. Raman Spectrosc.* **52**, 296–309 (2021).

53. A. Lombardi, M. K. Schmidt, L. Weller, W. M. Deacon, F. Benz, B. De Nijs, J. Aizpurua, J. J. Baumberg, Pulsed molecular optomechanics in plasmonic nanocavities: From nonlinear vibrational instabilities to bond-breaking. *Phys. Rev. X* **8**, 011016 (2018).

54. C. Carnegie, M. Urbieta, R. Chikkaraddy, B. De Nijs, J. Griffiths, W. M. Deacon, M. Kamp, N. Zabala, J. Aizpurua, J. J. Baumberg, Flickering nanometre-scale disorder in a crystal lattice tracked by plasmonic flare light emission. *Nat. Commun.* **11**, 682 (2020).

55. Y. Kim, S. Ji, J.-M. Nam, A chemist's view on electronic and steric effects of surface ligands on plasmonic metal nanostructures. *Acc. Chem. Res.* **56**, 2139–2150 (2023).

56. R. Chikkaraddy, J. Huang, D. Kos, E. Elliott, M. Kamp, C. Guo, J. J. Baumberg, B. De Nijs, Boosting optical nanocavity coupling by retardation matching to dark modes. *ACS Photonics* **10**, 493–499 (2023).

57. D. Thompson, J. Liao, M. Nolan, A. J. Quinn, C. A. Nijhuis, C. O'Dwyer, P. N. Nirmalraj, C. Schönenberger, M. Calame, Formation mechanism of metal–molecule–metal junctions: Molecule-assisted migration on metal defects. *J. Phys. Chem. C* **119**, 19438–19451 (2015).

58. BBI Solutions, Diagnostic Gold Colloid; https://www.bbisolutions.com/en/reagents/gold-colloid.

59. J. J. Baumberg, R. Esteban, S. Hu, U. Muniain, I. V. Silkin, J. Aizpurua, V. M. Silkin, Quantum plasmonics in sub-atom-thick optical slots. *Nano Lett.* **23**, 10696–10702 (2023).

60. G. Kresse, J. Hafner, *Ab initio* molecular dynamics for liquid metals. *Phys. Rev. B* **47**, 558–561 (1993).

61. J. P. Perdew, K. Burke, M. Ernzerhof, Generalized gradient approximation made simple. *Phys. Rev. Lett.* **77**, 3865–3868 (1996).

62. P. E. Blöchl, Projector augmented-wave method. *Phys. Rev. B* **50**, 17953–17979 (1994).

63. A. W. van der Vaart, *Asymptotic Statistics*, Cambridge Series in Statistical and Probabilistic Mathematics (Cambridge Univ. Press, 2000).

64. S. Sawyer, The Greenwood and exponential Greenwood confidence intervals in survival analysis; https://www.math.wustl.edu/~sawyer/handouts/greenwood.pdf.
